# Identifying adolescents with increased cardiometabolic risk—Simple, but challenging^☆^

**DOI:** 10.1016/j.jped.2024.10.001

**Published:** 2024-10-22

**Authors:** Eero A. Haapala

**Affiliations:** Sports and Exercise Medicine, Faculty of Sport and Health Sciences, University of Jyväskylä, Jyväskylä, Finland; Institute of Biomedicine, School of Medicine, University of Eastern Finland, Kuopio, Finland

The prevalence of increased cardiometabolic risk, such as insulin resistance and type 2 diabetes, has increased worldwide in adolescents.^1^ Insulin resistance and type 2 diabetes in childhood and adolescence predict insulin resistance, type 2 diabetes, and preclinical atherosclerosis^2^,^3^ as well as retinal and kidney disease^4^ in adulthood. In addition to the individual-level burden of these diseases, the economic costs of type 2 diabetes are measured in billions of US dollars.^5^,^6^ Therefore, to counteract these adverse effects and increased economic burden, the early identification of children and adolescents with increased risk of insulin resistance and type 2 diabetes is essential. However, objective methods, such as fasting blood samples or glucose tolerance tests, may not be commonly available or feasible at, for example, school healthcare centers due to the costs and the lack of appropriately trained laboratory staff. Therefore, indirect, affordable, and feasible indicators helping to identify youth with increased risk of cardiometabolic diseases are warranted.

Body composition, particularly body fat mass, and body fat percentage, is a well-recognized factor that influences the development of insulin resistance and subsequent risk of type 2 diabetes mellitus in children and adolescents.^7^ However, although dual-energy X-ray absorptiometry (DXA) is considered the reference method to assess body fat mass and body fat percentage in children and adolescents,^8^ body mass index and associated metrics, such as percentiles or standard deviation scores, remain the methods of choice for assessing overweight and obesity in youth.^9^ Even though the results of some studies suggest that body mass index is a feasible and as accurate method as DXA-derived body fat percentage in the prediction of cardiovascular mortality in adults^10^ and cardiometabolic risk in children,^11^ the use of the body mass index is widely criticized as it does not separate lean and fat mass or provide information about central obesity and may, therefore, misclassify children and adolescents with increased cardiometabolic risk.^12^ Therefore, body mass index alone may not provide the best available information and accuracy for the identification of individuals with increased cardiometabolic risk.

As body mass index alone may misclassify individuals with increased cardiometabolic risk, it is suggested that it should be used together with a measure of central adiposity to improve classification accuracy.^13^ The evidence suggests that waist circumference may have either independent or additive predictive value in identifying those with increased cardiometabolic risk in adults.^13^ In addition, the roles of body mass index and waist circumference in cardiometabolic health may also be paradoxical, as previous studies in adults suggest that at a given waist circumference, body mass index is associated with reduced cardiometabolic risk.^13^ While the exact mechanism for those observations is unknown, adipose tissue distribution, central vs. gluteal-femoral region, may explain these paradoxical findings.^13^ Moreover, body mass index may also be inadequate for monitoring the prevalence and development of overweight and obesity. There is some evidence that the positive news about the decelerated increase in the prevalence of overweight and obesity is shadowed by the increases in waist circumference.^14^ Therefore, it is necessary to better understand whether combining information from body mass index and waist circumference better identifies children and adolescents with increased cardiometabolic risk than either of these methods alone.

To fill the knowledge gap in adolescents, the article published in the *Jornal de Pediatria* by Bandeira et al.^15^ investigated the prevalence of insulin resistance and type 2 diabetes mellitus across different body mass index and waist circumference categories in 37,000 Brazilian adolescents. They found that the prevalence of increased insulin resistance was 53 % of adolescents in the overweight category defined by body mass index who also had high waist circumference and 70 % among those in the obesity category who had high waist circumference. In contrast, the prevalence of insulin resistance was substantially lower (32 % and 37 %, respectively) among adolescents with the same weight category but normal waist circumference. However, for type 2 diabetes mellitus, the prevalence increased with increasing body mass index category, almost irrespective of waist circumference. Furthermore, they also reported that increased insulin resistance was associated only with the obesity category and high waist circumference. In addition, the obesity category, irrespective of the waist circumference, was associated with an increased risk of pre-diabetes and type 2 diabetes mellitus. Conversely, adolescents with high waist circumference but normal body mass index did not have increased insulin resistance or higher prevalence of type 2 diabetes. These findings suggest that using the body mass index and waist circumference together improves the accuracy of discriminating against adolescents with increased cardiometabolic risk from other adolescents.

The results by Bandeira et al.^15^ among adolescents agree with the results from studies conducted in adults, suggesting that combining information from body mass index and waist circumference can improve the identification of individuals with increased risk of cardiometabolic diseases.^13^ As such, those results provide important information for clinicians to prevent and treat insulin resistance and type 2 diabetes mellitus in adolescents. Waist circumference, while providing additive information for identifying risk groups, may also be a feasible treatment target. Waist circumference is more responsive to treatment, such as physical activity or diet interventions, than body mass index.^16^, ^17^ Therefore, reducing waist circumference may provide significant cardiometabolic benefits, even without changes in body mass index. However, Bandeira and co-workers^15^ also observed that high waist circumference without increased body mass index was not associated with insulin resistance or type 2 diabetes. Therefore, a single measure of body adiposity may be insufficient to identify cardiometabolic risk accurately.

The next step in improving the identification of children and adolescents at increased risk of cardiometabolic diseases may include combining other measures than those related to body composition into the risk prediction models. For example, some evidence in children suggests that physical activity and sedentary time modify the ability of body adiposity to predict insulin resistance.^18^ Similarly, diet quality may modify the associations between body composition and insulin resistance.^19^ Therefore, to improve early identification beyond body composition and anthropometric measurements, holistic algorithms combining different measures of body composition and lifestyle factors could be developed and tested. Before such advancements, the results by Bandeira et al.^15^ provide important evidence that combining body mass index and waist circumference improves the accuracy of identifying adolescents at increased risk of cardiometabolic diseases.

In summary, the growing prevalence of insulin resistance and type 2 diabetes among adolescents represents a public health crisis with long-lasting consequences for both individuals and society. The limitations of existing screening tools, such as body mass index, highlight the need for better, more accurate methods of identifying adolescents at increased risk of cardiometabolic diseases. The study by Bandeira et al.^15^ has provided valuable evidence, suggesting that a combination of body mass index and waist circumference can improve the identification of adolescents with increased cardiometabolic risk. However, more research is needed to develop comprehensive algorithms for more accurate assessments.

## Conflicts of interest

The authors declare no conflicts of interest.
